# Female trail running: a systematic scoping review

**DOI:** 10.3389/fspor.2025.1694925

**Published:** 2026-01-19

**Authors:** Javier Espasa-Labrador, Øyvind Sandbakk, Álex Cebrián-Ponce, Alfredo Irurtia, Marta Carrasco-Marginet, John O. Osborne

**Affiliations:** 1 INEFC-Barcelona Research Group on Sport Sciences (GRCE), National Institute of Physical Education of Catalonia (INEFC), University of Barcelona (UB), Barcelona, Spain; 2 School of Sport Sciences, UiT The Arctic University of Norway, Tromsø, Norway

**Keywords:** female athletes, exercise physiology, menstrual cycle, athletic performance, sports nutrition, injuries

## Abstract

Trail running's popularity among women is increasing, yet research addressing the unique physiological demands, performance factors, and injury patterns in this population remains limited. This scoping systematic review aimed to map the existing research landscape on female trail runners; synthesize current evidence across physiological, nutritional, injury, and performance domains; and identify critical knowledge gaps to guide future investigations. A systematic search was conducted across four major databases (EBSCO, PubMed, Scopus, Web of Science) up to December 2024. Eligible studies were original peer-reviewed articles reporting sex-specific data on female trail runners within the above domains. Methodological quality was assessed using the Strengthening the Reporting of Observational Studies in Epidemiology (STROBE) checklist. Results from the 22 included studies (pooled sample ≈ 2,476 participants), predominantly published in the last decade, primarily focused on physiology and biomarkers (10 studies) and nutrition and body composition (6 studies), with fewer investigations into injuries (4 studies) or performance (3 studies). These studies indicated significant exercise-induced physiological stress and highlighted links between nutrition, body composition, and performance outcomes. However, a recurring limitation was the pervasive inconsistency in reporting participant characteristics, especially evident for key female-specific factors such as menstrual cycle status and hormonal contraceptive use, which were sparsely detailed. Furthermore, considerable heterogeneity in methodologies and the poor reporting of race characteristics and environmental conditions limited the synthesis of actionable insights. Most included studies (21 out of 22) demonstrated good methodological quality. In conclusion, while the research on female trail runners is growing, its practical application and the ability to draw robust conclusions are constrained by widespread reporting inconsistencies and a notable lack of depth in female-specific physiological data. Further progress in this field relies on the adoption of standardized reporting guidelines and a concerted effort to conduct robust, longitudinal investigations. Future studies should address hormonal influences, energy availability, effective training methodologies, and targeted injury prevention strategies tailored to female trail runners, ultimately to optimize their health, well-being, and athletic potential.

## 1 Introduction

Trail running is a form of off-road running in natural environments across diverse terrains, encompassing disciplines such as mountain, sky, and fell races, with no more than 20% of the route on paved roads (1, 2). Consequently, factors such as altitude, weather conditions, accumulated elevation, and gradient are critical variables in trail running (3–5). While success in endurance sport has been linked to high oxygen uptake (VO_2max_) and efficient running economy (6), the unique demands of trail running require more than just a well-developed steady-state aerobic capacity. Trail runners must continuously regulate their physical and psychological effort to navigate and respond to abrupt changes in gradient, technical terrain, and environmental stressors, while managing fatigue and injury risk. This regulation is highly individualized, as anthropometric characteristics and body composition significantly affect a runner's ability to meet these multidimensional challenges (3).

The popularity of trail running, particularly ultra-endurance running, has increased exponentially in recent years, and there has also been a simultaneous increase in female participation in the sport. For instance, female participation in the Western States 100-Mile Endurance Run rose from approximately 10%–12% in the late 1980s to approximately 20%–22% since 2001 (7). Globally, the number of women finishing 100 km ultramarathons grew by 44.9% between 2010 and 2019, and global race data show that female representation in trail events doubled from 13% to 26% between 2013 and 2019 (8). In some trail running distances, women now represent the majority of race finishers, compared to males. This trend of increasing female participation in the sport underscores the need for research specifically tailored to the physiological, biomechanical, and psychological demands faced by female trail runners (9).

Despite the anatomical, physiological, and biomechanical differences between male and female endurance runners being well-documented (10), the scientific community in the field of sports science has emphasized that most research has predominantly focused on male athletes (11). These differences likely necessitate sex-specific considerations for training, recovery, and performance optimization (12, 13). Current scientific evidence suggests that female endurance athletes have a different body size and composition compared to males, with higher absolute and relative fat mass, more subcutaneous fat mass, and lower lean mass (14). Furthermore, female endurance athletes have shown lower aerobic and anaerobic power, both in absolute values and normalized to their body mass, relative to men (15). However, they tend to metabolize fat more efficiently and may be more tolerant to cold environments, which may enhance trail running performance (16). In addition, sex hormone differences, particularly associated with the menstrual cycle, have been highlighted as a potentially important factor that could influence physical performance (17). These differences between men and women likely condition and differentiate the factors that influence performance in endurance races across both sexes. In addition to the contribution of physiological and biomechanical factors to performance in ultramarathon events, psychological factors (18) and injury incidence (19) must also be considered. Both are key elements for analyzing, understanding, and ultimately enhancing performance in this type of race.

Moreover, most current knowledge about performance factors originates from research on track or road disciplines, which do not capture the complexity and variability of trail running events. Factors such as uneven terrain, considerable altitude changes, and unpredictable weather conditions make trail running uniquely challenging, requiring specific adaptations that are often overlooked in studies on other forms of running. Considering these distinctions, the specific factors influencing performance in female trail runners remain largely unexplored. Given the increasing participation of women in this sport, there is a clear need to map the current evidence across key domains relevant to athlete health and performance.

A scoping review approach is well-suited to this emerging area, where heterogeneous methodologies and limited female-specific data limit systematic synthesis. Accordingly, this review aims to synthesize current evidence across four domains: physiological characteristics, nutrition and body composition, injury patterns, and performance outcomes. By identifying knowledge gaps and methodological limitations, this review seeks to provide direction for future research and support evidence-informed strategies to optimize the health, well-being, and performance of female trail runners (20).

## 2 Methods

This scoping systematic review explored the multidimensional factors influencing performance, injury incidence, and health outcomes in female trail runners, including physiological, biomechanical, psychological, and environmental aspects. The review followed the Preferred Reporting Items for Systematic reviews and Meta-Analyses—Extension for Scoping Reviews (PRISMA-ScR) guidelines (21) to ensure comprehensive reporting and methodological transparency. A review protocol was prospectively registered in PROSPERO (ID = CRD42021293231). The initial registration described the project as a systematic review; however, as the research evolved, the methodology was refined to a scoping review to better capture the breadth of evidence in this field and ensure alignment with the objectives of the study. These updates could not be made in PROSPERO, as the platform does not permit modification to certain key fields after registration. Accordingly, the original record has been retained, and all deviations are transparently reported in this manuscript. The protocol is publicly available at https://cutt.ly/nIaHlTJ.

### 2.1 Information sources and search strategy

A structured search strategy was employed across four databases: EBSCO, PubMed, Scopus, and Web of Science (WoS). These databases were chosen for their extensive indexing of peer-reviewed research in health and sport science, ensuring comprehensive coverage of relevant literature. The systematic search was completed on 31 December 2024. Titles, abstracts, and keywords were searched using the following terms and Boolean syntax: (“female”) AND (“trail” OR “off-road” OR “mountain” OR “ultra” OR “vertical kilometre” OR “fell” OR “ultraendurance” OR “sky”) AND (“run” OR “running” OR “marathon”). The same syntax was applied across all databases. Filters were set to include studies conducted on humans and published in English.

### 2.2 Eligibility criteria

Studies were included if they met the following criteria: (1) original, peer-reviewed articles; (2) data collected from one-stage competitive trail running events; (3) assessed physiological, performance-related, injury, psychological, or environmental factors; (4) included female trail runners in the study population; and (5) published in English. For this review, “one-stage” was defined as a single, continuous race, even if its duration extended over multiple days (e.g., 100-mile ultramarathons). Multistage races, in which athletes compete in discrete timed segments over several days, were explicitly excluded. Studies focusing on case reports, conference abstracts, editorials, commentaries, or reviews were excluded. No restrictions were applied regarding participants' physical fitness, race characteristics, or age. For studies that investigated specific events (e.g., Ultra-Trail du Mont-Blanc), the race characteristics (e.g., elevation gain and terrain) were evaluated against the definition of trail running described in the introduction. Only studies that reported sex-specific data for female runners were considered eligible. Studies that addressed deaths or hospitalizations were excluded as they did not fit the aim of this research.

### 2.3 Selection process

All search results were exported to a spreadsheet (Microsoft Excel 2021, Microsoft Inc., Seattle, WA, United States) for screening. One author (JE-L) conducted the initial database search and removed duplicates. Titles and abstracts were independently screened by two authors (JE-L and AP-C) to assess eligibility. Full texts of potentially relevant studies were reviewed against the inclusion criteria. Discrepancies were resolved through discussion, with arbitration by a third reviewer (AI) when required. Given the underrepresentation of female participants in sports science research (22), studies with mixed-gender samples were included if sex-disaggregated data for females were available.

### 2.4 Data collection process and data items

Data extracted from included studies encompassed as follows: (1) bibliographic information (e.g., authors, publication year, and journal); (2) research objectives; (3) study design and methodology; (4) participant characteristics (e.g., sample size, age, and fitness level); (5) study domains (e.g., physiological, performance, injury, psychological, and environmental); (6) primary outcomes and findings; and (7) study limitations. Two authors (JE-L and AP-C) independently extracted data, with discrepancies resolved through consensus or arbitration (AI). Extracted data were organized into structured tables (Microsoft Excel 2021). Due to heterogeneity in study designs and variables, a meta-analysis was not conducted. Variables were standardized where feasible (e.g., elevation and gradient) to facilitate comparisons.

### 2.5 Quality assessment and risk of bias

The quality and risk of bias of included studies were assessed using the Strengthening the Reporting of Observational Studies in Epidemiology (STROBE) checklist (23). Studies were categorized as (1) good quality (>14 points, low risk of bias); (2) fair quality (7–14 points, moderate risk of bias); or (3) poor quality (<7 points, high risk of bias). Two authors (JE-L and AP-C) conducted independent assessments, resolving disagreements through third-party evaluation (AI). The inclusion of diverse study designs necessitated a flexible approach to quality assessment, ensuring the rigor of this scoping review.

## 3 Results

### 3.1 Study selection

**Figure 1:**
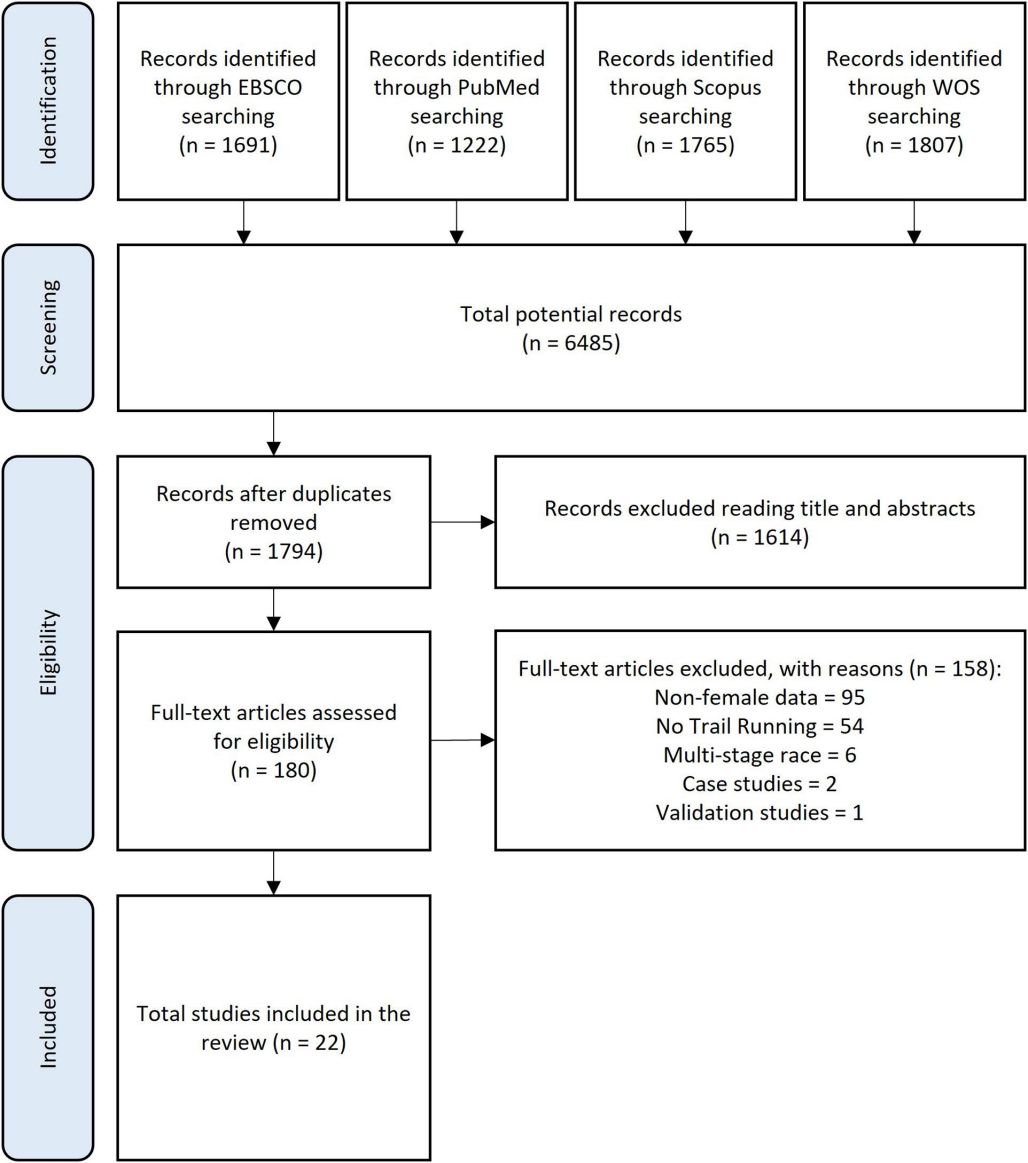
PRISMA-ScR flowchart.

Figure 1 summarizes all processes and results obtained by the search strategy. A total of 6,485 records were retrieved from the initial database searches, with 4,691 records identified as duplicates and removed. The remaining 1,794 publications were screened at the title and abstract level, with 180 publications progressing to the full-text screening stage. There were no disagreements between authors (JE-L and AP-C) for the inclusion or exclusion of studies. Full texts were retrieved and assessed against eligibility criteria, resulting in an additional 158 studies being excluded and leaving 22 studies for inclusion in the scoping review.

The primary reason for exclusion of publications in the full-text screen step was the absence of female-specific data. The topics and number of studies excluded at the full-text screening stage were as follows: non-female athletes or lack of sex-segregated data (*n* = 95); non-trail running (*n* = 54); multistage races (*n* = 6); and instrument validation studies (*n* = 1). Close to 80% (*n* = 17) of the included articles were published in the last decade (2014–2024), reflecting a growing interest in this field, particularly from 2018. Tables 1–4 present the variables and outcomes extracted from publications included in the qualitative synthesis analysis of this review.

**Table 1:** Data extracted from performance’s category publications.

Study	Participants characteristics			Variables	Outputs	Main conclusions
	*n*; age; level	Anthropometric: height; weight; BMI	Athletic: training/week; VO_2max_			
Hoffman (24)	18–29 years: *n* = 4	NR; NR; NR	NR; NR	Anthropometric and race time	Height (cm); mass (kg); BMI (kg/m^2^)	Participants in ultramarathon runs can vary widely in physical characteristics, with BMI values that would classify some individuals as underweight. Just among the top-five overall finishers, BMI still varied considerably ranging from 17.3 to 21.1 kg •m^–2^. Nevertheless, BMI was negatively associated with average running speed such that 10–11% of the variation in running speed was accounted for by BMI
	30–39 years: *n* = 19				Mean: 163.4 ± 7; 54.76 ± 6.06; 20.6	
	40–49 years: *n* = 41				18–29 years (*n* = 4): 166 ± 8; 57.2 ± 8.2; 20.7 ± 1.2	
	50–59 years: *n* = 13					
	>60 years: *n* = 5; NR				30–39 years (*n* = 19): 161 ± 7; 55.2 ± 6.7; 21.4 ± 1.5	
					40–49 years (*n* = 41): 164 ± 5; 54.7 ± 5.8; 20.4 ± 2.0	
					50–59 years (*n* = 13): 163 ± 6; 54.7 ± 6.1; 20.6 ± 1.5	
					>60 yeas (*n* = 5): 163 ± 9; 52.0 ± 3.5; 19.7 ± 1.4	
					Top five: NR; NR; 19.8 ± 1.5	
Knechtle et al. (9)	1,781; NR; NR	NR; NR; NR	NR; NR	Nationality of finishers, time trends in annual performance (mean time, fastest finisher’s time, etc.), and comparison between countries, changes in participation and performance	Some countries (e.g., men from Germany, Switzerland, Denmark, the Netherlands, Austria; women from the Netherlands or Germany) demonstrated a gradual change (slower or faster) in mean performance times.	Runners from Switzerland dominated both participation and performance in this high alpine ultramarathon. The authors suggest factors such as race prestige, prize money, local terrain familiarity, and possibly weather/language barriers may explain the rarity of East African athletes in such events. Among the limitations, the authors include the absence of training, anthropometric, and environmental data on the runners
					One Kenyan male runner (time ∼186% of the course record) ever participated, contradicting the initial hypothesis that Kenyan athletes—accustomed to altitude—would dominate	
Navalta et al. (25)	50; NR; NR	NR; NR; NR	NR; NR	Finish time and pacing race	Mean finishing time and pace (top finish time; 10th place finish time; top finisher pace; 10th place finisher)	Top athletes who compete in trail half-marathon races can expect to maintain their running velocity until approximately 40–49 years of age
					20–29 years: 1:48:19; 2:07:39; 8:34; 9:56	
					30–39 years: 1:51:22; 2:11:33; 8:27; 10:09	
					40–49 years: 1:50:35; 2:21:25; 8:53; 10:53	
					50–59 years: 2:15:45; 2:38:07; 10:14; 12:26	
					> 60 years: 2:47:06; 4:25:20; 14:13; 18:43	
Martinez-Navarro et al. (26)	13; NR; NR	NR; NR; NR	NR; NR	Demographic data, training data, lower limbs strength, and respiratory variables	Number of years running: 8 ± 3	Greater lower-limb isometric strength and enhanced ankle reactive strength emerged as two key determinants of mountain ultramarathon performance. Nevertheless, the absence of tests to assess lower-limb muscular endurance and not considering maximal oxygen uptake constrain the interpretation of these findings
					Number of races > 100 km: 2 ± 4	
					Weekly training days: 5 ± 1	
					Weekly running volume (km): 61 ± 13	
					Weekly positive elevation (m): 1,631 ± 565	
					Weekly training hours: 9 ± 5	
					Strength training (yes/no): 92%/8%	
					Leg Q-index: 1.72 ± 0.22	
					IMVC (N/kg): 10.32 ± 4.45	
					Squat jump height (cm): 21 ± 3	
					FVC (L): 3.65 ± 0.52	
					FEV1 (L): 2.97 ± 0.39	
					FEV1/FVC (%): 81.28 ± 2.63	
					PEF (L/min): 5.89 ± 1.39	
					MVV12 (L/min): 108 ± 23	
					MIP (cm H2O/kg): 1.39 ± 0.32	
Besson et al. (10)	18; 38 ± 6.6; NR	165.2 ± 6.6; 58 ± 7.2; 21.3	NR; 54.5 ± 5.5	Neuromuscular function, cost of running, blood parameters	Vastus lateralis RMS/M_max_ (%) (pre; post)	Women showed lower declines in knee extensor force and less contractile dysfunction in the plantar flexors than men after prolonged running, and sex-related differences in fatigability did not increase with race distance. Nonetheless, recruiting women for the study was challenging, even though the proportion of female participants (34%) exceeded the typical rate (e.g., ∼10% in events such as UTMB)
					Short race: 4.6 ± 1.2; 3.9 ± 1.2	
					Long race: 5.5 ± 2.3; 4.0 ± 3.0	
					Gastrocnemius RMS/M_max_ (%) (pre; post)	
					Short race:2.2 ± 1.0; 1.9 ± 1.0	
					Long race: 2.7 ± 1.8; 1.5 ± 0.8	
					Soleus RMS/M_max_ (%) (pre; post)	
					Short race: 2.2 ± 1.0; 2.4 ± 1.2	
					Long race: 2.8 ± 0.8; 2.3 ± 0.6	

**Table 2:** Physiology and biomarkers—extracted data.

Study	Participants characteristics			Variables	Outputs	Main conclusions
	*n*; age; level	Anthropometric: height; weight; BMI	Athletic: training/week; VO_2max_			
Salah (27)	12; 26.83 ± 6.81; NR	NR; NR; NR	NR; NR	Blood pressure, heart rate, and breath	Height (cm): 161 ± 4.99	Altitude of training, height, and BMI were significantly influential on athlete’s performance in high mountain competition whereas other anthropometric parameters such as weight, age, and physiological data as resting blood pressure, heart rate, and breathing rate do not influence performance
					Weight (kg): 60.63 ± 6.79	
					BMI (kg/m^2^): 23.45 ± 2.97	
					Systolic blood pressure (mmHg): 118.08 ± 9.63	
					Diastolic blood pressure (mmHg): 69.42 ± 6.65	
					Average blood pressure (mmHg): 48.67 ± 10.27	
					Mean arterial pressure (mmHg): 85.64 ± 6.08	
					Heart rate (bpm): 71.25 ± 12.02	
					Breathing rate (Mvt/min): 19.09 ± 2.26	
					Race time (s): 24,531 ± 2,588	
Copelan et al. (2014)	12 (4: 50 km, 8: 100 km); 47.1 ± 4.6 (premenopausal: 37.0 ± 5.4, postmenopausal: 57.2 ± 3.8); amateur	NR; NR; 23.7 ± 2.7	NR; 53.05 ± 3.8	Anthropometric, body composition, cardiorespiratory fitness, three blood samples (24 h pre, finish race, and 24 h post), and endocrine measurements	Premenopausal (pre-race; post-race; recovery):	Immediately post-race, estradiol levels showed a marked rise, averaging an increase of 97%. However, as a field-based observational study, exercise intensity and nutritional/hydration intakes could not be strictly controlled, thus limiting the interpretation of results
					Hematocrit (%): 39.7 ± 2.9; 37.2 ± 4.2; 33.9 ± 2.1	
					Postmenopausal (pre-race; post-race; recovery):	
					Hematocrit (%): 39.5 ± 2.9; 36.3 ± 7.0; 35.3 ± 7.1	
Carmona et al. (28)	1; 39.1; experienced	173; 67; NR	7.5 ± 3.5; NR	Average speed, CK (blood sample), cardiac troponin I (cTnI), muscle myosin isoform	Average speed: 7.26 km/h	There is evidence of muscle damage after prolonged mountain running, and an increase in SM serum concentration after a MUM could be indirect evidence of selective slow (type I) fiber-specific sarcomere disruptions
					Pre; post; 24 h; 48h:	
					CK (U/L): 233; 7,643; 5,819; 2,722	
					cTnI (ng/mL): 0,027; 0.260; 0.115; 0.101	
					Myosin: 988; 1,404; 6,999; 6,361	
Cote et al. (70)	8; 45.9 ± 10.2; amateur	162.7 ± 5.1; 58.9 ± 3.6; NR	NR; NR	Baseline baroreceptor sensitivity, heart rate variability, and arterial compliance; pre- and post-exercise echocardiographic evaluations of LV dimensions, volumes, Doppler flow velocities, tissue velocities, strain, and strain rate	Main finding related to fatigue in women’s participants (pre; post):	Both sexes exhibited comparable signs of exercise-induced cardiac fatigue after an ultramarathon. Higher baseline arterial compliance was associated with a lower degree of cardiac fatigue, as reflected by LV tissue velocities and strain. In addition, those with less ultra-running experience appeared more vulnerable to exercise-induced cardiac fatigue. However, the small sample size of female participants limited the generalizability of the findings
					LVEDV (mL): 100.9 ± 7.1; 87.8 ± 11.1	
					LVESV (mL): 34.0 ± 8.9; 30.4 ± 11.2	
					SV (mL): 66.9 ± 8.4; 57.4 ± 12.0	
					SVI (mL/m^2^): 41.2 ± 6.3; 35.2 ± 7.1	
					FS (%): 42.5 ± 6.5; 34.6 ± 7.9	
					E/A: 1.55 ± 0.51; 1.30 ± 0.27	
					IVRT (ms): 89.8 ± 11.7; 73.5 ± 19.4	
					Ar duration (ms): 133.4 ± 25.9; 119.6 ± 12.0	
Temesi et al. (71)	10; 41 ± 10; experienced	164 ± 4; 57 ± 6; NR	NR; NR	Demographic data, torque of knee extensor and plantar flexors, and EMG	Finish time (hh:mm:ss): 21:53:32 ± 2:43:04	In a mountain ultra-trail race, women experienced less objectively measured fatigue, reflected by a smaller MVC loss in the knee extensors and reduced peripheral fatigue in the plantar flexors compared to men. Notably, this study included 95% of all female race finishers.
					Maximal and evoked torques (pre; post):	
					MVC KE (N·m): 115 ± 27	
					TwPot KE (N·m): 37 ± 6	
					Db100 KE (N·m): 61 ± 12	
					Db100 KE (N·m): 59 ± 9	
Chlíbková et al. (29)	3; 39.7 ± 5.7; experienced	169.8 ± 1.7; 59.9 ± 4.9; 20.7 ± 1.3	13.0 ± 1.0; NR	Anthropometric measurements (height, body mass), hematocrit, hemoglobin, electrolytes (Na+, K+), markers of muscle/renal function (CK, creatinine, blood urea, estimated GFR), fluid intake, supplements, and menstrual cycle phase and race’s performance data (time, distance, finishing place)	Body mass slightly increased in one case (possible overdrinking) but decreased in the other two. All three athletes showed mild hyponatremia (133–134 mM) at the end of the race. Two of them exhibited CK concentrations greater than 10,000 U/L, but without overt kidney dysfunction. Fasting pre-race parameters (e.g., creatinine and hematocrit) were within normal ranges, and no remarkable post-race renal impairment was observed	Three experienced female ultra-athletes, each winning a different 24 h race in diverse weather conditions and disciplines, all developed mild post-race hyponatremia. This suggests that close monitoring of fluid and electrolyte balance is critical, even for well-trained and successful endurance athletes. Additionally, one participant’s slight body mass gain underscores the need for caution regarding potential overhydration. The authors emphasize the significance of hormonal status and training history in mitigating or exacerbating fluid- and muscle-related stresses
Deneen and Jones (30)	25; mid 20s to 50+; recreational	NR; NR; NR	NR; NR	Hormonal biomarkers (salivary cortisol, salivary alpha-amylase). Sampling time points (pre-, mid-, and post-race for distances up to 100 km). Race performance (distance, finish times). Demographic data (sex, age), though the study focused primarily on biomarker changes	Cortisol and alpha-amylase showed significant changes during the ultra-running event. Cortisol levels generally rose during the race, reflecting activation of the hypothalamic–pituitary–adrenal (HPA) axis. Salivary alpha-amylase also varied in response to physiological stress, consistent with sympathetic nervous system activity.	Prolonged running (beyond the marathon distance) induces marked shifts in stress-hormone levels. Both cortisol and alpha-amylase responded to the increasing demands of ultra-running, underscoring the roles of the HPA axis and autonomic nervous system in coping with extreme physiological stress. The findings highlight the complexity of hormonal regulation over extended distances and suggest that monitoring these biomarkers may be a useful indicator of an athlete’s stress response during ultra-endurance races
					Some runners exhibited suppressed salivary alpha-amylase at distances beyond the marathon, though overall patterns varied	
Maqueda et al. (72)	3 (82 km: 2; 14 km: 1); mean: 35.6 ± 2.2, 82 km: 38.75, 14 km: 37.6; experienced	Mean: 168 (82 km: 168.5, 14 km: NR); mean: 61.7 (82 km: 63.5, 14 km: NR); NR	Mean: 11.7 (82 km: 15, 14 km: 5); NR	Blood, RNA, and microarrays	Nearly one-fourth of all protein-coding genes were affected by an ultra-mountain trail race.	Nearly one-fourth of all protein-coding genes were impacted by an ultra-mountain trail (UMT), highlighting a significant representation of multiple human biological pathways. Nevertheless, interpretations were constrained by a small sample size and the possibility of varying effort levels among runners completing the same distance
					Numerous human biological pathways were significantly overrepresented after the race, reflecting broad transcriptional changes	
Chlíbková et al. (31)	6; 37.7 ± 11.9; NR	182.6 ± 8.0; NR; NR	9.0 ± 5.4; NR	Anthropometric measures. Pre-race experiences. Blood samples. Total water, plasma urea, creatinine, and CKs	Pre; post:	After the 24 h winter mountain race, participants experienced a reduction in body fat alongside elevated markers of muscle damage and renal stress. Notably, changes in skeletal muscle correlated with total body water fluctuations. However, the study was constrained by measuring limb circumferences only once—potentially affecting muscle estimates—and using bioelectrical impedance analysis (BIA), instead of isotope dilution, to evaluate total body water
					Body mass (kg): 68.0 ± 67.4; 67.9 ± 5.8	
					Body fat (kg): 17.9 ± 5.4; 16.6 ± 5.5	
					Body fat (%): 26.4 ± 7.2; 24.3 ± 6.9	
					Skeletal muscle mass (kg): 27.7 ± 3.7; 28.6 ± 3.2	
					Total body water (L): 37.2 ± 3.5; 37.1 ± 4.3	
					Plasma urea (mmol/L): 4.6 ± 1.1; 8.1 ± 2.3	
					Plasma creatinine (*μ*mol/L): 64.7 ± 9.6; 69.0 ± 15.6	
					Creatine kinase (U/L): 118.9 ± 36.6; 2,009 ± 2,248	
					Creatinine clearance: 114.4 ± 27.4; 110.4 ± 32.0	
Williams et al. (84)	54; NR; mixed-experienced	NR; NR; NR	NR; NR	Effect of biosynchronous music on trail running performance, comparison between cadence-synchronous and heart rate-synchronous music, perceived effort using the Borg scale (6–20), and distance covered and running pace	Cadence-synchronous music resulted in faster running pace for both men and women and heart rate-synchronous music led to slower pace than cadence-based synchronization. Males ran significantly faster than females across all conditions. Females using heart rate-synchronous music ran significantly longer than any other group. Males using cadence-synchronous music ran significantly longer than those using heart rate-synchronous music. Males using cadence-synchronous music covered the longest distances. Female runners using heart rate-synchronous music ran longer than their male counterparts. Cadence-based music lowered perceived effort in men. Heart rate-based music lowered perceived effort in women	Biosynchronous music influences running performance and perceived effort, with cadence-synchronous music enhancing speed and distance in men, while heart rate-synchronous music reduces perceived exertion and increases run duration in women. These findings suggest optimizing music interventions based on training goals and gender differences. Limitations: lack of anthropometric/fitness data, small sample size, self-reported effort bias, and uncontrolled environmental factors
Collado et al. (32)	18; 41 ± 6; amateur	161 ± 5; 56.92 ± 4.36; NR	9.07 ± 2.54; NR	Hematological, hormonal, body composition, and strength.	Pre; post; 24 h; 48h:	Higher endogenous basal testosterone concentrations were associated with greater muscle strength in female ultra-endurance runners. Nevertheless, the study’s interpretation was limited, first, by a lack of uniformity in hormonal contraceptive (HC) regimens (type, dose, and route of administration), and, second, by a relatively small sample size
					Estradiol (pg/mL): 89.06 ± 87.99; NR; NR; NR	
					Testosterone (ng/dL): 19.34 ± 9.34; NR; NR; NR	
					T/E ratio: 1.25 ± 1.99; NR; NR; NR	
					CK (UI/L): 137.59 ± 54.73; 5,075.75 ± 3,871.18; 2,036.61 ± 1,389.83; 905.3 ± 534.95	
					LDH (UI/L): 185.88 ± 25.29; 380.59 ± 112.80; 320.93 ± 93.97; 303.38 ± 86.67	
					MM (%): 39.20 ± 3.45; 38.00 ± 4.86	
					HG (kg): 31.33 ± 3.69; 28.67 ± 4.10	
					SJ (cm): 20.76 ± 2.72; 18.47 ± 2.38	
Martinez-Navarro et al. (26)	13; 42 ± 6; NR	NR; NR; NR	NR; NR	Cardiorespiratory variables (VO_2max_, forced vital capacity, maximal flow volume loop), strength (squat jump and hand grip)	BMI (kg/m^2^): 21.7 ± 2	Expiratory function decreased around mid-race, with a further decline later, echoing changes in relative running speed. Overall, both pulmonary and inspiratory muscle function were moderately reduced post-race, but women demonstrated a more pronounced FEV1/FVC drop, suggesting greater lower airway obstruction. Interpretation was limited because pulmonary function measures were volitional
					VO2peak (mL O2/min/kg): 51.5 ± 5.2	
					Vpeak (km/h): 14.4 ± 1.4	
					VVT1 (km/h): 10.1 ± 0.9	
					VVT2 (km/h): 12.5 ± 1.3	
					Number of years running 8 ± 3	
					Number of races > 100km: 2 ± 4	
					Weekly training days: 5 ± 1	
					Weekly running volume (km): 61 ± 13	
					Weekly positive elevation (m): 1,631 ± 565	
					Weekly training hours: 9 ± 5	
					Strength training (yes/no): 92%/8%	
					Mean (pre; post)	
					FVC (L): 3.49 ± 0.48; 3.08 ± 0.81	
					FEV(L): 2.77 ± 0.42; 2.03 ± 0.61	
					FEV1/FVC (%): 78.67 ± 4.66; 67.75 ± 16.9	
					PEF (L/s): 6.05 ± 1.33; 4.84 ± 1.96	
					MIP (cm H2O): 78 ± 20.9; 63 ± 20	
Vauthier et al. (73)	9; 43.94 ± 8.95; mixed-experienced leve (ITRA points: 144 to 914)	NR; NR; NR	NR; NR	Glomerular filtration rate assessed by serum creatinine, classified according to the RIFLE (risk, injury, failure, loss, end-stage kidney disease) criteria. Time points of measurements: baseline, end of each of six loops (every 26 km), race end, and after 24 h of rest	Risk of acute kidney injury measured via RIFLE classification:	The study found significant deterioration in renal function among ultra-trail runners, particularly during the initial stages of the race (around the marathon distance, approximately 52 km). Acute kidney injury, defined as moderate according to the RIFLE classification, occurred in 41.7% of participants, predominantly in the early stages of the event, and was notably associated with increased dropout rates for reasons unrelated to musculoskeletal injuries. Although the authors reported that 25% of female runners presented renal risk, no significant gender-specific conclusions were highlighted. Key limitations included small sample size, logistical challenges during data collection, reliance on serum creatinine (potentially influenced by muscle damage), and absence of osmolarity measures to confirm hydration status
					Overall (modified intention-to-treat analysis, mITT): 32.7% presented at least one “risk” (RIFLEr) event.	
					Peak risk moment at 52 km (∼marathon distance) with 29.6% classified as “risk.”	
					Per protocol analysis: 41.7% presented at least one RIFLEr event.	
					Peak risk again at 52 km, with 37.1% classified as “risk.”	
					Average GFR values (PP analysis): baseline: 96.55 ± 12.90 mL/min/1.73 m²	
					Lowest GFR at 52 km: 74.51 ± 13.01 mL/min/1.73 m²	
Burger et al. (33)	1; 42.9 ± 8.0; amateur	NR; NR; NR	NR; NR	Cardiac function parameters assessed by echocardiography:	No separate analyses or results specifically for the female runner were presented.	Participation in an ultra-endurance marathon (130 km) resulted in a transient decrease in cardiac function, specifically affecting the right ventricle, in athletes with elevated levels of cardiac biomarkers (hs-cTnI and NT-proBNP). Athletes exhibiting substantial biomarker elevations had significant reductions in both left and right ventricular function immediately and five days post-race. However, these cardiac alterations did not occur in athletes without substantial biomarker elevations. Limitations include the small sample size, particularly the underrepresentation of female runners (only one participant), limiting generalizability and necessitating confirmation in more diverse and larger cohorts
				Left ventricular ejection fraction (LVEF)	Significant reduction in right ventricular fractional area change (FAC) immediately after the ultramarathon (48.0 ± 4.6% vs. 46.7 ± 3.8%, *p* = 0.011), persisting 5 days after the race (48.0 ± 4.6% vs. 46.3 ± 3.9%, *p* = 0.027).	
				Right ventricular FAC		
				Global longitudinal strain (GLS)		
				Cardiovascular biomarkers:		
				High-sensitivity cardiac Troponin I (hs-cTnI)		
				N-terminal pro-brain natriuretic peptide (NT-proBNP)	No significant changes in LVEF were observed overall.	
					Athletes with biomarker elevations (hs-cTnI and NT-proBNP) above the median had significant reductions in both LVEF and FAC, both immediately and five days after the event	
Cebrián-Ponce et al. (74)	37; 14 km: 35.1 ± 11.4 years, 35 km: 35.5 ± 6.7 years and 52 km: 34.3 ± 3.8 years; mixed-experienced level	14 km: 165.5 ± 6.4, 35 km: 164.2 ± 6.6, and 52 km: 163.9 ± 3.7 cm; 14 km: 63.0 ± 9.5, 35 km: 56.5 ± 6.0, 52 km: 55.1 ± 3.3; NR	NR; NR	Whole-body bioelectrical impedance vector analysis (WB-BIVA):	Women showed significant body mass reductions after all race distances: 14 km: −0.9 ± 0.7% (*p* < 0.001)	The study highlights significant dehydration and muscle damage in trail runners, with larger physiological changes observed at greater race distances. Specifically, women showed pronounced muscle damage (CK elevation) and significant dehydration post-race in medium (35 km) and long (52 km) distances, but not significantly at the shortest (14 km). Despite these clear physiological changes, hydration status showed a complex, not strictly linear relationship with race performance. Limitations included uncontrolled food and fluid intake, a small sample size for muscle-localized analysis (only men included), and heterogeneous participant fitness levels. Bioelectrical impedance vector analysis (BIVA) was effective in capturing these body composition changes, suggesting its potential for monitoring hydration and muscular status in endurance sports
				Resistance adjusted for height (R/H), reactance (Xc/H), vector length (Z/H), and phase angle (PhA)		
					35 km: −1.3 ± 1.9% (*p* = 0.006)	
					52 km: −1.8 ± 1.3% (*p* = 0.039)	
					CK levels increased significantly post-race:	
					14 km: 81.6 ± 63.4% increase (*p* = 0.002)	


					35 km: 156.3 ± 115.3% increase (*p* = 0.001)	
					52 km: 433.5 ± 244.5% increase (*p* = 0.068;	
					marginally significant due to small sample size)	
					WB-BIVA analysis showed dehydration tendencies (increased impedance values), significantly pronounced for women in the 35 km (*p* < 0.001) and 52 km (*p* < 0.001) groups, but not significantly in the 14 km women group	
				Muscle-localized bioelectrical impedance vector analysis (ML-BIVA) was performed only in a male subgroup (*n* = 11), not including females. CK: marker of muscle damage		
Drigny et al. (34)	12; 45.2 ± 13.5; experienced	NR; NR; NR	NR; NR	Achilles tendon cross-sectional area, Achilles tendon echogenicity, medial gastrocnemius muscle pennation angle, thickness, length, and fiber length.	Baseline Achilles tendon (AT) CSA was significantly smaller in women compared to men (56.57 mm² females vs. 73.10 mm² men; *p* < 0.001)	This study demonstrated significant biphasic adaptations of Achilles tendon architecture during a 156 km mountain ultramarathon. Initially, a decrease in Achilles tendon cross-sectional area (AT CSA) was observed, reflecting immediate mechanical stress, followed by a secondary increase, possibly indicating intratendinous edema as an adaptive response, which persisted at least 12 h post-race. These tendon adaptations correlated with running speed and spatiotemporal running characteristics. Gender differences at baseline included smaller AT CSA and lower muscle thickness (MT) in women, potentially affecting running biomechanics and mechanical efficiency. Limitations include the relatively small number of female participants, uncontrolled environmental conditions, and the influence of frequent measurement interruptions. The findings highlight the complex nature of tendon and muscle adaptive responses during prolonged trail running, warranting further studies focused on sex-specific analyses and longer follow-up periods for recovery assessment
				Performance parameters: speed, ground contact time, stride length, flight ratio, and running/walking ratio	Echogenicity of AT was significantly higher in women compared to men (129.0 vs. 115.1, *p* = 0.010), indicating differences in tendon structural characteristics between genders.	
				Occurrence of lower-limb pain		
					MT at baseline was lower in women compared to men (15.71 mm vs. 17.82 mm; *p* = 0.034).	
Stewart et al. (75)	10; 41 ± 9; experienced	NR; NR; NR	NR; NR	Alveolar-capillary function and lung diffusion measured by lung diffusing capacity for carbon monoxide, alveolar membrane conductance, and capillary blood volume.	Women-specific results were not separately presented.	The study concluded that ultramarathon trail running results in transient impairments in lung diffusion capacity, primarily driven by reductions in pulmonary capillary blood volume (Vc) and alterations in alveolar-capillary membrane conductance (Dm) during exercise. Mild cardiac stress (as indicated by biomarkers) and lung fluid accumulation were also observed, highlighting transient cardiovascular and pulmonary stress. Limitations included a limited female sample size (*n* = 10, 15%), preventing gender-specific conclusions, and varied race conditions possibly influencing physiological responses. The findings suggest further studies with increased female representation and controlled environmental conditions are warranted to better understand gender-specific physiological adaptations and risks in ultramarathon runners
					General significant findings:	
					Increase in cardiac biomarkers post-race (e.g., cTnI: pre 0.04 ± 0.02 vs. post 0.13 ± 0.03 ng·mL^−^¹; BNP: pre 20 ± 2 vs. post 112 ± 21 pg·mL^−^¹; *p* < 0.01).	
				Cardiac biomarkers: troponin I (cTnI) and brain natriuretic peptide (BNP).		
				Lung fluid accumulation and cardiac function (stroke volume and cardiac output)	Significant post-race increase in lung comet tails indicates mild-to-moderate lung fluid accumulation.	
					Reduction in resting and exercise stroke volume post-race (76 ± 2 mL to 69 ± 2 mL, *p* < 0.01).	
					Reduction in lung diffusion capacity post-race (DLco and Vc reduced; Dm reduced only during exercise)	
Tsai et al. (76)	1; 37; experienced	NR; NR; NR	NR; NR	Cardiac autonomic regulation evaluated by heart spectrum blood pressure monitors to detect arrhythmias.	Postural hypotension: observed in 44.4% of participants immediately after the race (the female participant did not present postural hypotension).	The study observed significant acute changes in cardiac autonomic regulation immediately following a 246 km mountain ultramarathon. Notably, postural hypotension and exercise-induced premature ventricular complexes (PVC) were common findings, with faster running speeds associated with a higher incidence of PVC. All participants evaluated showed marked increases in cardiac biomarkers (hs-TnT and NT-proBNP) post-race, indicating physiological cardiac stress rather than myocardial injury. Limitations included a very small sample size and only one female participant, greatly limiting the generalizability of these findings, particularly for female runners. Future studies with larger cohorts, especially involving more female athletes, and continuous monitoring during and after events, are necessary to comprehensively understand the cardiovascular impacts in ultra-endurance athletes
					Premature ventricular complex (PVC): detected post-race in 33.3% of participants in a standing position, including the female participant.	
				Postural hypotension measurement		
				Cardiac biomarkers:	Cardiac biomarkers:	
				High-sensitivity troponin T (hs-TnT),	Female-specific values were not separately detailed; however, the female participant was included among the six finishers who underwent biochemical assessments	
				NT-proBNP		

**Table 3:** Nutrition and body composition—extracted data.

Study	Participants characteristics			Variables	Outputs	Main conclusions
	n; age; level	Anthropometric: height; weight; BMI	Athletic: training/week; VO_2max_			
Hoffman (35)	82; top-five women: 41 ± 4; experienced	Men: 176.1 ± 7.2; 71.9 ± 8.7; 23.2 ± 2.2	NR; NR	BMI and its correlation with performance. Comparison of anthropometric characteristics between sexes and age groups. Association between body weight, height, and running speed	BMI and Performance Correlation:	This study demonstrates the wide variation in physical characteristics among ultramarathoners. Despite this variability, BMI was negatively correlated with running speed, explaining 10%–12% of the variation in performance. The findings suggest that a slightly higher BMI than in marathon runners may be beneficial for ultramarathon performance, possibly due to greater muscle mass and fat reserves, which could aid in endurance over long distances and rough terrain. While lighter runners tend to be faster, BMI alone does not fully determine performance, as many other factors (e.g., training volume and metabolic efficiency) play a role in ultramarathon success. Lack of physiological data (VO_2max_, body fat percentage, training history), no assessment of body composition (lean mass vs. fat mass), and a single-race study (Western States Endurance Run), limiting applicability to other trail ultramarathons with different conditions were the main limitations reported
					Negative correlation between BMI and running speed (*r*² = 0.11, *p* < 0.0001 for men; *r*² = 0.10, *p* = 0.02 for women).	
		Women: 162.2 ± 7.3; 54.8 ± 6.5; 20.6 ± 1.7				
					Lower BMI values were associated with faster running times.	
					Anthropometric Variability:	
					Significant differences in height, weight, and BMI between men and women.	
					Wide BMI distribution among participants, including some in the underweight (<18.5) and overweight (>25) categories.	
					Comparison with other runners:	
					BMI values of top female ultramarathoners (19.8 ± 1.5 kg/m²) were comparable to elite marathoners.	
					Male ultramarathoners had a higher BMI (23.2 kg/m²) than typical elite marathon runners (19–21 kg/m²)	
Hoffman (24)	20–64; NR; NR	NR; NR; NR	NR; NR	Anthropometric and body composition	BMI (kg/m^2^): 21.2 ± 2.1 (mean), 21.5–22.9 (top 3 finishers). Body fat (%): 21 ± 6 (mean), 19 ± 6 (finishers), 24 ± 4 (non-finishers)	This study demonstrates that there are wide variations in BMI and percent body fat among participants in 161 km ultramarathon runs
Moran et al. (83)	1; 25; recreational	156.5; 48; 19.6	NR; NR	Macronutrient, fluid, and sodium consumption, gastrointestinal tolerance and food preferences during the race, the impact of nutrition on performance, and perceived effort	Energy intake:	This case study highlights the importance of individualized nutrition strategies for ultra-endurance athletes. The athlete shifted from sweet to savory foods during the race, emphasizing the role of varied textures and flavors in maintaining intake, preventing flavor fatigue, and sustaining energy. Sports drinks and broth were key for hydration and electrolyte balance. The study underscores the need for flexible nutrition plans, allowing adaptation to food preferences and race conditions, with support teams playing a crucial role in ensuring proper intake. Findings are limited to a single subject, with no control over external conditions (temperature, terrain), and the athlete was a first-time ultramarathon participant, limiting comparability with habitual strategies
					10,890 kJ (736 kJ/h)	
					558 g of carbohydrates (44 g/h)	
					7,403 mg of sodium (500 mg/h, 52 mmol/L)	
					6,150 mL of fluids (415 mL/h)	
					Carbohydrate intake variation:	
					First half of the race: 34 g/h	
					Second half of the race: 53 g/h	
					Fluid sources and distribution:	
					Gatorade Endurance (sports drink): 3,850 mL consumed, contributing 189 g carbs, 2,700 mg sodium, and 2,835 kJ	
					Cola drink: 850 mL consumed, providing 91 g carbohydrates and 83 mg caffeine	
					Savory broth: provided the highest sodium content (3,000 mg, 41% of total intake)	
					Gastrointestinal tolerance and food preferences:	
					The athlete did not report any gastrointestinal discomfort. Initially relied on sweet foods but shifted their preference to savory foods later in the race. Preferred high-sodium fluids and snacks after experiencing mild cramping	
Melo et al. (36)	16; 29.5 ± 7.6; elite	158.9 ± 6.2; 53.37 ± 3.64; 20.89 ± 1.53	NR; NR	Anthropometric, body composition, somatotype, cardiorespiratory capacity, heart rate, blood pressure, and oxygen saturation	Mass (kg): 53.37 ± 3.64	The results seem to show differences between male and female mountain runners in anthropometric characteristics and cardiorespiratory capacity and that anthropometric characteristics can influence the cardiorespiratory performance of mountain runners. The main constraint lies in the limited number of participants and a lower number of female athletes
					Height (cm): 158.94 ± 6.18	
					BMI (kg/cm^2^): 20.89 ± 1.53	
					∑ 8 folds (mm): 108.53 ± 24.14	
					Fat mass (%): 18.18 ± 3.52	
					Bone mass (%): 29.97 ± 3.29	
					Muscle mass (%): 39.93 ± 3.31	
					Somatotype (endo; meso; ecto; weight index): 6.50 ± 8.99; 4.07 ± 0.80; 2.33 ± 0.92; 42.23 ± 1.27	
Ishihara et al. (37)	3; 42.6 ± 1.2; 158.0 ± 6.5	47.9 ± 3.8; 18.9 ± 0.7; NR	NR; NR	Running speed, glucose, and diet	Glucose during running (average; SD; highest; lowest):	A higher carbohydrate intake was associated with faster running speeds, but the athletes’ top blood glucose levels were not linked to running velocity. Instead, maintaining glycemic control—rather than rapid carbohydrate availability—seems vital for performance. Suboptimal carbohydrate consumption might contribute to blood glucose declines observed in ultramarathons. Nevertheless, the study’s interpretation was limited by a small number of participants and the use of flash glucose monitoring rather than blood sampling
					124; 19.03; 182.67; 82	
					Running speed (min/km): 5.31	
					Energy intake (kcal/kg/h): 4.03	
					Carbohydrate intake (g/kg/h): 0.84	
					Protein intake (g/kg/h): 0,06	
					Fat intake (g/kg/h): 0.041	
					Carbohydrates consumed per product	
					type (g/kg/h):	
					Liquids and gels: 0.55	
					Fruits and sweets: 0.046	
					Solids: 0.24	
Bouscaren et al. (77)	525; NR; NR	165 (160–170); 56 (52–61); 20.7 (19.6–22.0)	NR; NR	Pre-race survey: demographic characteristics, training experience, experience in hot environments, medical history, illness, heat acclimatization, and cooling strategies	Pre-race survey:	A large proportion of runners (approximately one-quarter) reported not using any hydration plan under warm and humid conditions. The remaining three-quarters followed a predefined approach, targeting roughly 650 mL/h. However, as this was a survey-based study, the primary limitation arises from potential self-report and recall biases
					Lower prevalence of cramps in women.	
					24.8% declared had trained in the heat.	
					65.2% of women declared early arrival to the race place for acclimatizing to local environmental conditions.	
				Post-race survey: impact of environmental conditions on performance, heat-related symptoms, and hydration	Post-race survey:	
					Heat-related symptoms were reported by 46.8% of female runners. Women planned to ingest 624 ± 229 mL/h.	
					Only 24% of women planned to drink sodium-enriched water	
Ishihara et al. (38)	1; 44; professional	1.52; 42; 18.2	13.8; NR	Blood glucose fluctuations during a 155.7 h ultra-endurance event, nutritional intake patterns (solid food vs. liquid/gels), macronutrient contributions (carbohydrates, proteins, fats). Impact of nutrition on performance (running pace correlations) and serum biomarkers (total protein, triglycerides, liver enzymes)	Running pace was positively correlated with carbohydrate (*p* = 0.01) and energy intake (*p* = 0.02). No significant correlation was found between glucose level and running speed (*p* = 0.79). Triglycerides decreased significantly (140 mg/dL to 33 mg/dL). Total protein levels dropped (6.9 g/dL to 5.8 g/dL). CK levels increased from 102 to 1,312 U/L, indicating muscle damage. LDH levels rose from 184 to 428 U/L, suggesting sustained muscle stress	Solid food consumption contributed to a more stable blood glucose level and better maintenance of running speed than liquid/gels. Protein intake helped prevent excessive blood glucose drops. A multiday continuous ultra-endurance race does not result in extreme glucose fluctuations, even with severe sleep deprivation. As a limitation, the authors highlighted that this research was a single-subject design with a lack of a control group, and the terrain and physiological responses introduced uncontrolled variables
Parent et al. (78)	12 (21.8%); 43.5 ± 9.3; experienced (ITRA points: 530.7 ± 63.5)	165 ± 6; 53.9 ± 5.5; NR	61.7 ± 38.8 km; NR	Glycemic excursions through continuous glucose monitoring before, during, and after a 156 km ultra-trail race.	Female-specific results were not reported separately for metabolic, hormonal, or glycemic outcomes. General findings applicable to all runners (males and females):	This study demonstrated that a 156 km ultra-trail race did not significantly increase the risk of hypo- or hyperglycemia during running itself but resulted in significant hyperglycemia during the 48 h post-race recovery period. Hyperglycemia appeared related to muscle damage and associated inflammation, as indicated by elevated levels of creatine phosphokinase (CPK) and lactate dehydrogenase (LDH). Despite these substantial physiological disturbances, glycemic fluctuations did not significantly affect immediate physical performance or behavioral alertness. Major limitations include the loss of precise nutritional intake data and the lack of separate analysis for female athletes. The findings suggest strategies mitigating muscle damage or inflammation might help prevent post-race hyperglycemia and protect vascular health in athletes participating repeatedly in ultra-endurance events
					No increased risk of hypoglycemia or hyperglycemia observed during the active running periods.	
				Metabolic markers (glucose, insulin, cortisol, free fatty acids, *β*-hydroxybutyrate)	Significant hyperglycemia during the 48 h recovery period post-race, with 31.9% of runners showing glycemic values >180 mg/dL.	
					Exercise intensity correlated positively with hyperglycemic episodes.	
					Marked increase in muscle damage markers (CPK and LDH) during and after the race (CPK 43.6 times higher at 24 h post-race vs rest).	
				Muscle damage indicators (creatine phosphokinase—CPK, LDH)		
				Exercise intensity (heart rate reserve percentage: HRR%)		
				Behavioral alertness (reaction time, lapses of attention, sleepiness)		
Valder et al. (79)	Study A: 3 (17.6%), Study B: NR; Study A: 26 ± 4, Study B: NR; Study A: recreational, Study B: NR	Study A: NR; NR; NR	Study A: NR; NR	Intestinal barrier (IB) functionality markers: serum endotoxin (LPS), intestinal fatty acid-binding protein (i-FABP), soluble CD14, and interleukin 6 (IL-6). The effect of consuming diluted cloudy apple juice (polyphenol-rich) vs. a sugar-only placebo drink and water after exercise	Study A:	Moderate endurance exercise caused transient increases in intestinal permeability markers, which normalized rapidly post-exercise. Consumption of sugar-rich beverages post-exercise did not significantly impair intestinal barrier recovery in moderate exercise but negatively impacted intestinal barrier recovery after ultra-endurance activity. This negative effect was mitigated by consuming a polyphenol-rich fruit juice matrix, indicating protective potential. Limitations included a very small female participant number, the absence of specific female data analyses, and the lack of controlled dietary conditions during ultramarathon conditions. Future research should incorporate larger, balanced gender groups and more comprehensive dietary standardization to confirm these findings
		Study B: NR; NR; NR	Study B: NR; NR		Increased serum endotoxin, i-FABP, CD14, and IL-6 immediately post-run (*p* < 0.05). Levels of these markers returned rapidly to baseline within 180 min post-exercise. No significant impact of test beverages (polyphenol-rich juice or sugar drink) on intestinal permeability markers, except a minor difference in CD14, suggesting possible immune-modulatory effects of the apple juice matrix.	
					Study B (not reported women’s participation):	
					dramatic increase in serum endotoxin and IL-6 immediately post-race (IL-6 increased approximately 20-fold compared to moderate exercise).	
					Quick recovery of intestinal barrier (endotoxin levels) observed after race in water and apple juice groups, but was significantly impaired in the placebo (pure sugar) group.	
					The polyphenol-rich juice reduced endotoxin levels similar to water, suggesting protective effects against sugar-induced intestinal permeability	
Vilar et al. (80)	13 (40.6%); 42.2 ± 1.7; amateur	NR; NR; NR	10 ± 0.9; NR	Oxidative stress markers: glutathione peroxidase, glutathione reductase, malondialdehyde, carbonyl groups	Specific results were not separated by gender; combined group results were:	The study concluded that beetroot supplementation during a 107.4 km ultra-endurance mountain trail positively affected post-race recovery, significantly improving oxidative status and reducing muscle damage, indicated by lower CK and LDH levels and reduced loss of muscular strength. However, beetroot supplementation showed no significant effect on inflammatory response (CRP). Limitations included the small sample size, particularly in beetroot-consuming athletes (only 6 participants total, 3 females), hindering robust statistical power and gender-specific conclusions. Further controlled intervention studies with larger samples, including more female runners, are recommended to confirm these findings and clarify the effectiveness of beetroot supplementation for recovery in ultra-endurance sports
					Oxidative status:	
					GPx significantly increased in beetroot consumers at 24 and 48 h post-race (125.3 ± 17.4 vs. 91.7 ± 4.9 µmol/L × min at 24 h; *p* < 0.05).	
				Muscle damage indicators: creatine kinase, lactate dehydrogenase		
				Inflammation markers: C-reactive protein		
				Muscular strength: assessed by the squat jump test	Lower lipid (MDA) and protein (CG) oxidative damage post-race in beetroot consumers.	
					Muscle damage:	
					Significantly lower CK and LDH at the finish line for beetroot consumers (CK: 2,071.7 ± 406.7 IU/L vs. 5,616.01 ± 816.1 IU/L, LDH: 299.4 ± 20.7 IU/L vs. 390.6 ± 22.4 IU/L, *p* < 0.05)	
					Inflammation: no significant impact of beetroot consumption on CRP levels post-race.	
					Muscular strength: loss of strength (SJ) at the finish line significantly less in beetroot consumers compared to non-consumers	

**Table 4:** Injuries—extracted data.

Study	Participants characteristics			Variables	Outputs	Main conclusions
	n; age; level	Anthropometric: height; weight; BMI	Athletic: training/week; VO_2max_			
González-Lázaro et al. (39)	555; NR; NR	NR; NR; NR	NR; NR	Races’ characteristics and runners injured (%)	The races’ mean distance was 28 ± 6 km, and their mean accumulative positive/negative elevation change was 3,497 ± 717 m. The maximum elevation was 1,810 ± 371 m.	The incidence of musculoskeletal injuries during 20–42 km mountain running races is low. The majority of injuries experienced by runners are minor in nature and located in the lower extremities, mainly the ankles. Only musculoskeletal injuries were collected, while medical illness or skin disorders were not considered
					The total number of injured participants was 28. 21% of female runner participants suffered any injury	
Gajardo-Burgos et al. (81)	241 females;>18; mixed-level	NR; NR; NR	NR; NR	Injury details (anatomical location and type) and illness details (type)	Injured athletes: 74	Almost one-third of trail runners reported injuries, and nearly one-quarter reported illnesses during the event. Gradual onset injuries affecting 11% were related to non-running activities. The knee, ankle, and foot were most affected. Limitations included recall bias and self-diagnosis, though self-reported methods are generally reliable. Some athletes misunderstood instructions regarding training load reporting. Multidisciplinary teams could use these findings to enhance education strategies and medical support
					Non-injured athletes: 167	
					Illness athletes: 59	
					Non-illness athletes: 182	
					Injury’s location (*n*; %):	
					Neck: 0;0/shoulder: 1; 1.4/thoracic spine: 0;0/Lumbosacaral: 5; 7.2%/pelvis: 3; 4.3%/hip/groin: 10; 14.3%/thigh: 8; 11.4%/knee: 21; 30%/Lower leg: 6; 8.6%/ankle: 8; 11.4%/	
					Foot: 8; 11.4%	
					Illness type (*n*; %):	
					immunological: 5; 8.1%/respiratory: 46; 74.2%/gastrointestinal: 5; 8.1%/others: 3; 4.8%/musculoskeletal: 3; 4.8%	
Matos et al. (40)	190; NR; amateur	NR; NR; NR	NR; NR	Demographic and injury data	Time in trail running (1–4 years; 5–8 years; >9 years): 163; 25; 2	The lower limbs are the most often affected body region, with dermatological injuries [blisters, irritation (chafing), and superficial wounds] being the most frequent type of injury. The retrospective design makes it impossible to generalize the results
					Injuries derived from training/competition: 85.5%	
					Injury rate: 9.62 per 1,000 h	
					Injured athletes: 163	
					Injury’s location (*n*; %):	
					Hip: 40;7,2%/cervical spine: 7;1.3%/dorsal spine: 5;0.9%/lumbar spine: 22;4%/thigh (anterior area): 22;4%/thigh (posterior area): 28;5.1%/thoracic zone: 3;0.5%/leg injuries: 39;7%/knee: 108;19.5%/ankle: 66;11.9%/toes: 38; 6.9%/toenails: 155;28%/Ears: 5;0.9%/other: 16;2.9%	
					Types of injuries (*n*;%):	
					Blisters: 148;22.4%/sprains: 66;10%/irritation: 108;16.3%/superficial wound: 75 (11.3%)	
Viljoen et al. (41)	92; mean: 37.3 (38 km: 34.8, 65 km: 37.8, 100 km: 37.8); NR	NR; NR; NR	NR; NR	Demographic, training experience, and injury data	Total average and per race (38, 65, 100 km)	Trail runners training for the 2019 SkyRun reported RRIs that mostly affected the lower limb, specifically the knee, ankle, and foot. A study that used self-reported injury data based on injuries that occurred during the previous 12 months
					Height (cm): 167.1; 168.3; 167.7; 166.2	
					Weight (kg): 61.9; 66.4; 62.5; 59.6	
					BMI (kg/m^2^): 24.6; 24.6; 22.2; 21.6	
					Retrospective annual incidence (RRIs) (*n*; %):	
					Lower limb: 26; 72.2%/ankle: 8; 22.2/knee: 7; 19.4/foot: 5; 13.9	
					Muscle and tendon injuries (*n*; %): 17; 16.7	
Viljoen et al. (41)	59 (36%): 46 km: 39 (54%), 80 km: 10 (20%), 161 km: 9 (28%), 322 km: 1 (14%); 40.9 ± 1.0; mixed-experienced level	NR; NR; NR	NR; NR	Injury rate (injuries per 1,000 h of running).	The injury rate was significantly higher in shorter distances (46 km = 3.09 injuries/1,000 h) compared to longer distances (80 km = 0.68; 161 km = 1.09 injuries/1,000 h).	Ultra-trail runners training for shorter ultradistances (46 km) had significantly higher injury rates, greater injury diversity, and more severe injuries compared to runners preparing for longer distances. Most injuries were located in the lower limb, involving muscle and tendon pathologies. Notably, shorter-distance participants, predominantly females, showed more diverse and severe injuries, possibly due to less experience and different training characteristics. Limitations include the retrospective self-reported design and potential underreporting of injuries. Future research should focus on prospective data collection and more detailed analysis of factors influencing injury risk across different race distances, especially considering female-specific data
				Point prevalence of injury (3 weeks before the race).		
				Injury severity (days missed from training/racing).		
				Injury characteristics by anatomical region, body area, tissue type, and pathology		
					Point prevalence of injury: 0% (likely underreported due to fear of disqualification).	
					Injury severity: higher severity among shorter-distance groups (46 km: 13.2 ± 18.4 days missed; 80 km: 16.4 ± 9.2 days missed).	
					Characteristics of injury:	
					The lower limb is most frequently injured (89%).	
					Most injuries involved muscles/tendons (56%), particularly muscle injuries (31%) and tendinopathies (25%). Only the 46 km participants (with the highest female representation) reported diverse injury profiles, including head, upper limb, and trunk injuries, as well as more severe injuries such as fractures	
Zapata-Rodrigo et al. (82)	36; NR; international level	168 ± 8; 62 ± 9; 21.2 ± 2.1	7–8; NR	Injury and illness frequency, injury details (anatomical location, type, and mechanism) and illness details (organ system involved and likely etiology), and severity (time-loss or performance-limiting classification	Incidence: 38% of the 77 athletes had an injury or illness during the WMTRC 2023: 22% sustained a musculoskeletal injury (the majority new on race day, 82%), 16% developed an illness (e.g., hyperthermia and dehydration). Injury profile: foot was the most frequently affected location (47% of reported injuries) followed by ankle and knee (12% each). The most common mechanisms included direct contact with objects (roots, stones) or slip-and-fall, friction/blister formation, and overuse. About illness, thermoregulatory symptoms (hyperthermia or dehydration) accounted for 67% of all cases, and digestive or nutritional issues were also reported.	Approximately 38% of these elite trail runners incurred injuries or illnesses at the WMTRC 2023, with foot injuries most frequent and dehydration/hyperthermia the main causes of morbidity. Approximately 12% withdrew, often due to severe dehydration, acute trauma (e.g., fractures/dislocations), or exacerbation of chronic injuries. However, findings are limited by the small sample (two national teams), possible selection bias toward healthy top-tier athletes, and data from only one championship; plus, the lack of systematic anthropometric/fitness measurements (e.g., VO₂_max_) restricts broader applicability
					Impact on performance: 24% of all athletes had an injury/illness that temporarily limited their participation (from minutes to not be able to participate). Among those who were injured/ill, 30% of injuries and 33% of illnesses led to withdrawal	

### 3.2 Fields of research of the studies

A total of 22 studies were included in this scoping review, categorized into four domains: physiology and biomarkers, nutrition and body composition, injuries, and performance. This organization was adopted to facilitate analysis and provide a comprehensive framework to summarize the body of research within this scoping review. Table 5 summarizes the included articles.

**Table 5:** Summary of included studies by domain.

Study	Domain	Design	Race(s) name(s) (distance, country)	STROBE
Hoffman (35)	Per	Descriptive cross-sectional	Way Too Cool (50 km, United States); American River (80 km, United States); Western States Endurance Run (161 km, United States)	Good (18)
Hoffman (24)	N and BC	Descriptive cross-sectional	Western States Endurance Run (161 km, United States)	Good (18)
Hoffman (35)	N and BC	Descriptive cross-sectional study	Rio del Lado Endurance Run (161 km, United States)	Good (17)
Moran et al. (83)	N and BC	Case study	The North Face 100 (100 km, Australia)	Fair (13)
Knechtle et al. (9)	Per	Descriptive retrospective study	Swiss Alpine Marathon (78 km, Switzerland)	Fair (14)
Copeland et al. (69)	Phy	Prospective observational study	NR (50 km, 100 km, and 100 miles, NR)	Good (18)
Carmona et al. (28)	Phy	Experimental study	Cavalls del Vent (85 km, Spain)	Good (16)
Coates et al. (3)	Phy	Observational longitudinal study	Fat Dog 100 UTR (100 and 160 km, Canada)	Fair (14)
Temesi et al. (71)	Phy	Comparative experimental study	North Face® Ultra-Trail du Mont-Blanc (110 km, Switzerland, Italy, and France)	Good (18)
Chlíbková et al. (29)	Phy	Descriptive cross-sectional study	Adidas 24 h (116km: 10 km/lap, Czech Republic)	Fair (13)
Deneen and Jones (30)	Phy	Descriptive cross-sectional study	Pumpkin Holler Hunnerd (10 km, 25 km, and 100 km, United States)	Good (19)
Maqueda et al. (72)	Phy	Experimental laboratory study	Cavalls del Vent (85 km, Spain)	Good (18)
Navalta et al. (25)	Per	Retrospective observational study	Moad Trail Half Marathon (21 km, United States)	Fair (14)
Chlíbková et al. (31)	Phy	Descriptive cross-sectional study	Adidas 24 h Open Championship of the Czech Republic in Winter Mountain Ultra-Marathon (11.4 km/lap, Czech Republic)	Fair (14)
Melo et al. (36)	N and BC	Descriptive cross-sectional study	N/A	Fair (14)
González-Lazaro et al. (39)	Inj.	Retrospective longitudinal study	Alto Sil (32 km, Spain); Desafío Urbión (36 km, Spain); Integral del Valdecebollas (42 km, Spain)	Good (17)
Ishihara et al. (37)	N and BC	Descriptive cross-sectional study	Ultra-trail Fuji (165 km, Japan)	Good (17)
Martinez-Navarro et al. (26)	Per	Descriptive cross-sectional study	Penyagolosa (107.4 km, Spain)	Good (18)
Williams et al. (84)	Phy	Randomized experimental study	NA (4.7 km/lap, United Kingdom)	Fair (14)
Besson et al. (10)	Per	Comparative observational study	Martigny-Combe à Chamonix (40 km, Switzerland and France); Orsières-Champex-Chamonix (56 km, Switzerland and France); Courmayeur-Champex-Chamonix (101 km, Switzerland and France); Sur les Traces des Ducs de Savoie (145 km, Italy and France); Ultra-Trail du Mont-Blanc (171 km, Italy, Switzerland and France)	Good (18)
Bouscaren et al. (77)	N and BC	Descriptive cross-sectional study	La Diagonale des Fous (165 km, France); Le Trail de Bourbon (111 km, France); La Mascareignes (65 km, France)	Good (18)
Collado-Boira et al. (32)	Phy	Cross-sectional observational study	Penyagolosa Trail CSP (107.4 km, Spain)	Good (18)
Gajardo-Burgos et al. (81)	Inj.	Retrospective cohort study	NR (11 km, 18 km, 24 km, 45 km, and 63 km, Chile)	Good (18)
Ishihara et al. (38)	N and BC	Case study	Shiga Round Trail (438 km, Japan)	Good (19)
Martinez-Navarro et al. (26)	Phy	Descriptive cross-sectional study	Penyagolosa (107.4 km, Spain)	Good (17)
Viljoen et al. (41)	Inj.	Retrospective cross-sectional study	SkyRun (38 km, 65 km, and 100 km, South Africa)	Good (18)
Vauthier et al. (73)	Phy	Prospective descriptive study	Trail Scientifique de Clécy (156 km: 26 km/lap, France)	Good (19)
Burger et al. (33)	Phy	Prospective descriptive study	Rundumadum Ultra-Marathon (130 km, Austria)	Good (19)
Cebrián-Ponce et al. (74)	Phy	Descriptive observational study	Volta a la Cerdanya Ultrafons (14 km, 35 km, and 52 km, Spain)	Good (17)
Drigny et al. (34)	Phy	Descriptive longitudinal study	Trail Scientifique de Clécy (156 km: 26 km/lap, France)	Good (19)
Kishi et al. (85)	Psy	Descriptive cross-sectional study	La Diagonale des Fous (165 km, France); Le Trail de Bourbon (111 km, France)	Fair (14)
Niering et al. (86)	Psy	Descriptive cross-sectional study	NA (>42 km, Germany, United States, Great Britain, Austria, and Spain)	Good (17)
Parent et al. (78)	N and BC	Descriptive longitudinal study	Trail Scientifique de Clécy (156 km: 26 km/lap, France)	Good (18)
Daniel et al. (87)	Psy	Descriptive cross-sectional study	Brazil 135 Ultramarathon (217 km, Brazil)	Fair (14)
Stewart et al. (75)	Phy	Longitudinal observational study	Hong Kong 100 (103 km, Hong Kong), Courmayeur-Champex-Chamonix (101 km, Italy, Switzerland, and France); Ultra-Trail du Mont-Blanc (171 km, France, Italy, and Switzerland)	Good (19)
Tsai et al. (76)	Phy	Longitudinal observational study	Run Across Taiwan Ultra-Marathon (246 km, Taiwan)	Good (18)
Valder et al. (79)	N and BC	Experimental study	TorTour de Ruhr® Ultra-Marathon (160.9 km and 230 km, Germany)	Fair (14)
Vilar et al. (80)	N and BC	Observational longitudinal study	Penyagolosa Trails CSP® (107.4 km, Spain)	Fair (14)
Viljoen et al. (41)	Inj.	Retrospective cross-sectional study	Mac Ultra Races (46 km, 80 km, 161 km, and 322 km, South Africa)	Good (19)
Zapata-Rodrigo et al. (82)	Inj.	Cross-sectional observational study	Long Trail (86 km, Austria); Short Trail (45 km, Austria); Mountain Classic (15 km, Austria), Vertical (7 km, Austria); Under-20 Classic (15 km, Austria)	Good (18)

N and BC: nutrition and body composition; Per, performance; Psy, psychology; Inj, injuries; Phy, physiology.

The physiology and biomarkers domain encompassed studies investigating physiological processes and responses in female trail runners, including biomarkers of muscle damage [e.g., creatine kinase (CK) and myoglobin], hormonal responses, and neuromuscular fatigue. The nutrition and body composition domain included studies evaluating dietary strategies, macronutrient intake, and the relationship between anthropometric characteristics and performance. The injuries domain comprised of studies reporting the incidence, location, and types of injuries observed in trail runners, as well as factors contributing to injury prevention and recovery. The performance domain focused on metrics directly related to race outcomes, such as completion times, pacing strategies, and strength parameters that influence endurance.

Most studies (*n* = 10) focused on physiology and biomarkers and explored topics such as cardiac function, endocrine responses, pulmonary adaptations, and muscle architecture changes during or after ultramarathons. Studies on nutrition and body composition (*n* = 6) examined feeding strategies, carbohydrate requirements, and body composition changes in various ultramarathon contexts. Research on injuries (*n* = 4) evaluated musculoskeletal injuries, injury rates across training and competition phases, and specific injury patterns related to ultramarathons. Finally, studies on performance (*n* = 3) analyzed sex differences in trail running outcomes, environmental influences, and neuromuscular fatigue.

### 3.3 Participants, races, and characteristics of the studies

Significant inconsistencies were observed in the reporting of participant characteristics across the included studies. Several studies did not disaggregate data by sex, and few provided detailed information on female participation, such as age, anthropometrics, performance level, training experience, or key physiological parameters (e.g., VO_2max_). In addition, very few publications reported participants' menstrual cycle phases or hormonal contraceptive use.

**Figure 2:**
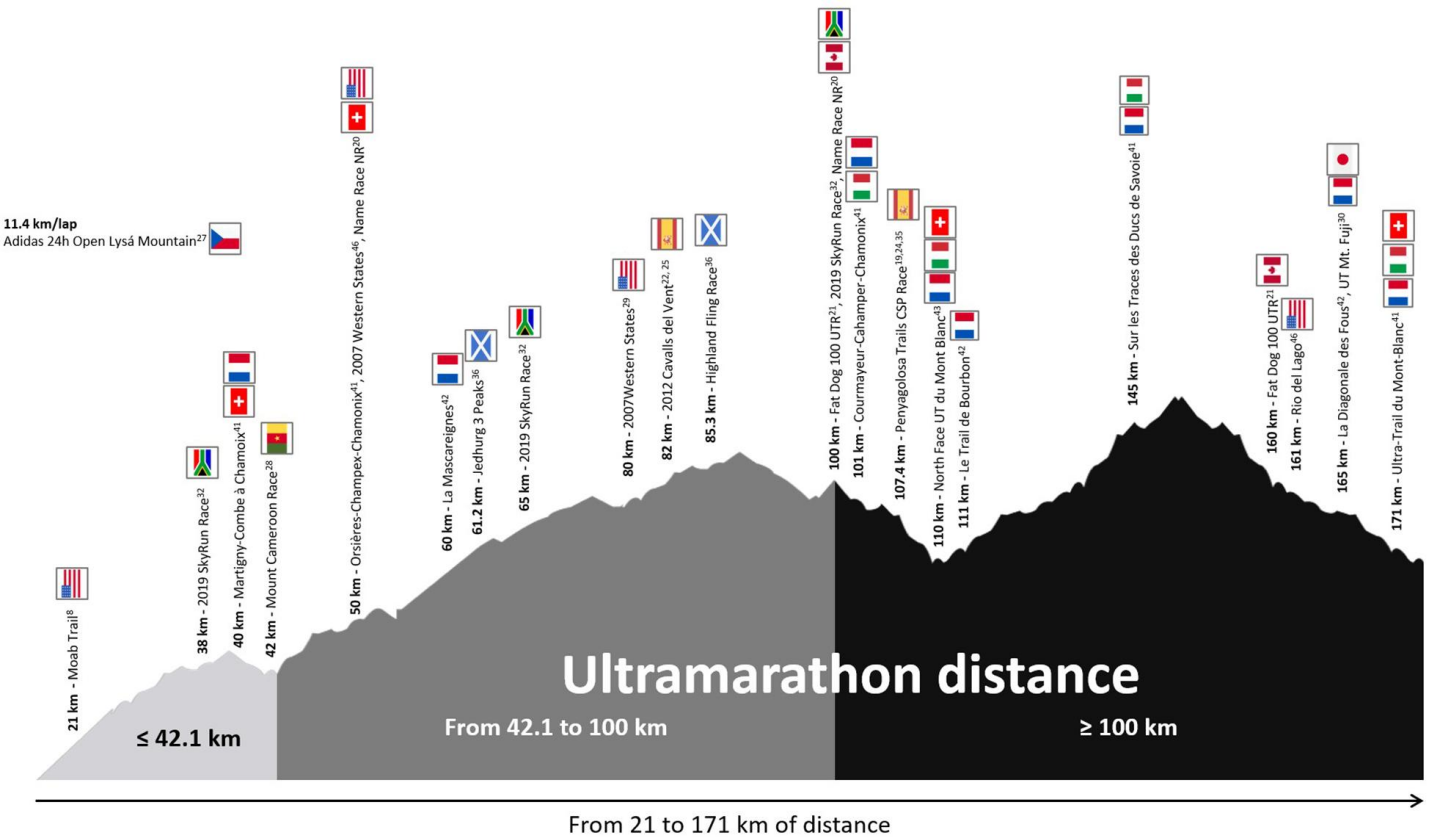
Race distances and countries represented in the included studies in this review.

Figure 2 shows all the studies included, which covered a wide range of race distances, with several studies investigating multiple ultramarathon distances (e.g., events ranging from 38 to 171 km). Common race distances reported included 82 and 107.4 km, 100 miles, and 165 km, or renowned races such as the Ultra-Trail du Mont-Blanc. Unique race formats were also represented, such as a 24 h race conducted on an 11.4 km loop. A smaller number of studies investigated shorter trail races, including a 42 km mountain race, a trail half-marathon, or collections of several official mountain races.

### 3.4 Domains of research: topics and outcomes

The physiology and biomarkers domain encompassed a diverse range of parameters. Frequently assessed biomarkers included creatine kinase, cardiac troponin, and cortisol, measured pre- and post-race. Other biomarkers, such as serum ferritin, myoglobin, and inflammatory cytokines, were reported less consistently. Four studies evaluated cardiac function, reporting changes in ventricular compliance and myocardial recovery following ultramarathons. Furthermore, three studies examined respiratory muscle strength and ventilatory efficiency, identifying sex differences in respiratory performance and its relationship to endurance outcomes. A unique aspect reported by one study was the ribonucleic acid (RNA) changes and gene expression modulation following a mountain ultramarathon, which provided novel insights into muscle repair mechanisms. Detailed information on these physiological parameters and biomarkers can be found in Table 1.

In the nutrition and body composition domain, seven studies reported on variables such as total body fat, lean mass, and skinfold thickness. Some studies detailed nutritional strategies, addressing macronutrient intake during races, hydration practices, and the effect of specific supplementation (e.g., vitamin D_3_ and polyphenols) on recovery and oxidative stress mitigation. These findings underscored the importance of individualized nutritional strategies tailored to the demands of ultramarathon events. Data related to the nutrition and body composition domain are presented in Table 3.

The injuries domain included four studies, which collectively reported an overall injury incidence of 32% among female trail runners. The lower limbs were consistently identified as the most commonly injured anatomical regions. These studies focused on the prevention of overuse injuries, biomechanical contributors to injury risk, and strategies for reducing acute injury incidence during training and competition. Related data are summarized in Table 4.

Finally, research categorized under the performance domain explored sex differences in running velocity, pacing strategies, and endurance duration. These studies also analyzed ultramarathon pacing strategies, which emphasized the influence of gradient, terrain variability, and environmental conditions on pacing and overall performance. Additionally, one study investigated lower-limb strength and its association with reactive performance and endurance. Data from these performance studies are summarized in Table 4.

### 3.5 Quality assessment and risk of bias of the studies included

Among the 22 included studies, 21 were rated as “good quality,” and one study was rated as “fair quality.” No studies included were identified as of poor quality. Table 5 details the specific STROBE score for each publication.

## 4 Discussion

To the best of the authors' knowledge, this is the first scoping systematic review to comprehensively map and synthesize existing research across the multidimensional domains influencing female trail runners. This review, which ultimately included 22 studies, revealed a distinct distribution in research focus across the identified domains. The primary finding was that the physiology and biomarkers domain was the most extensively investigated area (*n* = 10), with these studies investigating a broad range of parameters such as muscle damage biomarkers (e.g., creatine kinase and myoglobin), hormonal responses, neuromuscular fatigue, cardiac function, endocrine responses, pulmonary adaptations, and muscle architecture changes during or after ultramarathons. The next most common domain was nutrition and body composition (*n* = 6), which assessed dietary strategies, macronutrient intake, the relationship between anthropometric characteristics and performance, feeding strategies, carbohydrate requirements, and body composition changes in various ultramarathon contexts. The injury domain was addressed in four studies, reporting on injury incidence, anatomical location, and types, as well as factors such as injury prevention, recovery, injury rates across training and competition, and specific injury patterns. Finally, the performance domain was the least studied (*n* = 3), with investigations reporting aspects such as race completion times, pacing strategies, strength parameters, sex differences in trail running outcomes, the influence of environmental factors, and neuromuscular fatigue. This distribution highlights that while research into female trail running is increasing, the literature to date has disproportionately focused on the physiological and nutritional domains, with comparatively limited research on injury and performance outcomes.

A key aspect highlighted by this review is the apparent discrepancy between the high methodological quality scores, with 21 of 22 studies rated as “good” according to the STROBE checklist, and the pervasive omission of reporting key female-specific variables. This paradox emphasizes an important limitation of standardized quality assessment tools, such as STROBE, when applied to research involving female athletes. Although invaluable for evaluating general reporting quality, STROBE does not include specific items that address critical variables such as menstrual cycle status or hormonal contraceptive use. This omission is not trivial, as it may significantly confound the interpretation of results across physiology, performance, and injury domains. For example, hormonal fluctuations across the menstrual cycle can directly influence aspects such as substrate utilization, thermoregulation, and markers of muscle damage and recovery. Consequently, the absence of reported female-specific information in the reviewed studies makes it unclear whether the findings are generally applicable or specific to a particular hormonal state.

This issue reflects the broader systemic bias in sports science, where research protocols and reporting frameworks remain predominantly designed around male participants. Failure to integrate female-specific variables continues to constrain scientific progress and limits the translation of research evidence into real-world practice. Our findings confirm and demonstrate that this gap persists in trail running research, where basic descriptors for female participants, such as hormonal status, are seldom reported. To meaningfully advance trail running research and ensure that future studies are both inclusive and relevant, there is a clear need to develop and adopt consensus-based reporting guidelines for research on female trail runners, modeled on the broader recommendations proposed by Elliott-Sale et al. (42).

### 4.1 Participant and race characteristics

A significant challenge encountered in synthesizing the literature for this review was the inconsistent reporting of participant characteristics across the 22 included studies. Notably, several studies failed to provide sex-disaggregated data, and many offered limited information on female participants' age, anthropometrics, performance level, training history, or key physiological parameters such as VO_2max_. This methodological limitation is reflective of the broader issue of underrepresentation and insufficient reporting of female-specific data within sports science research (11), which curtails the ability to draw robust, generalizable conclusions for the female trail running population. It is evident that there needs to be improved, standardized reporting for female trail running research, particularly as this scoping review highlights that trail running attracts a highly heterogeneous cohort of female athletes.

Despite these reporting inconsistencies, available data indicate the mean age of female trail runners was 37.1 ± 5.2 years, with a typical age range of 30 to 47 years. This aligns with existing literature suggesting that endurance sports are commonly undertaken by older populations (25); however, younger female runners (<30 years), including elite athletes, were also present in this scoping review data. To capture the full demographic spectrum and developmental trajectories within trail running, future research should actively seek to include and analyze data from these younger participants across diverse competitive levels. Similar heterogeneity was apparent in the reported anthropometric and physiological profiles. For instance, mean body mass index (BMI) values ranged from 18.9 to 23.7 kg·m^−^² and mean VO_2max_ values varied from 53.1 to 54.5 mL·kg¹·min^−^¹. Both BMI and VO_2max_​ are acknowledged as important predictors of trail running performance (26, 35, 36), with higher body mass-normalized VO_2max_ typically correlating with superior endurance capacity (26). The wide variance in these physiological values within the reviewed literature likely reflects the frequent co-participation of both amateur and professional athletes in trail running events, leading to diverse physiological data in the study samples (29). Critically, relatively few studies reported on key female-specific variables, such as menstrual cycle phase or hormonal contraceptive use. This omission represents a significant data gap that warrants urgent attention and must be addressed in future research, given the potential influence of hormonal and menstrual status on performance, recovery, and injury risk in female trail runners.

The race characteristics of the included studies were similarly diverse. Event distances ranged from shorter trail events (e.g., half-marathon and marathon mountain races) to ultramarathons of up to 171 km. These events featured wide variability in cumulative ascents (2,300–10,300 m), descents (2,743–4,356 m), maximum altitudes (1,280–4,090 m), and ambient temperatures (ranging from −20.6 to 19.9°C), as detailed in Table 5. However, most studies provided only limited descriptive information about specific race conditions, typically restricted to total distance or starting temperature, with minimal detail on the fluctuations of key environmental factors (e.g., wind speed, humidity, or temperature) throughout the events.

Environmental changes, a common phenomenon in ultradistance trail running events, especially those at high altitude, can potentially affect both athlete performance and athlete safety. Thermal stress (e.g., cold, heat, and humidity), changes in atmospheric pressure, and altered air composition (e.g., altitude or pollution) are known to influence endurance performance (43). For example, a 1°C decrease in core body temperature has been associated with: (a) a ∼5% decreased aerobic performance due to lower VO_2_, attributed to a 10–30 beats per minute decrease in maximum heart rate; (b) decreased strength and power; and (c) impaired manual dexterity due to numbness and loss of sensitivity (44, 45). In extreme cases, the environmental conditions may pose a risk to health, particularly when combined with a high level of fatigue, which may lead to technical errors during the race in the often-remote locations of trail running events (2). It is crucial that future research prioritizes detailed and systematic collection of environmental data at multiple relevant time points (e.g., pre-race, mid-race, and post-race), with the aim of understanding how changes in ambient conditions may influence the performance and athletes' health in each part of the race and its cumulative effect.

To meaningfully advance scientific knowledge and improve translation of research into practice, future trail running research should adopt standardized methodologies for reporting participant characterization and race profiling. This would include comprehensive descriptions of anthropometric and body composition data, training history, validated performance levels, and, importantly, consistent reporting of menstrual cycle status and hormonal contraceptive use, which are all factors crucial to understanding female physiology and performance, particularly in the context of ultramarathon events (2). Adopting a more precise and uniform categorization of runners based on individual characteristics, competitive level, and specific race profiles will allow for clearer inter-study comparisons and the generation of more robust, practically applicable findings. Encouragingly, existing scientific guidelines offer robust frameworks that can, and should, be adopted to enhance the methodological rigor, transparency, and consistency of future research in this rapidly evolving and increasingly popular domain of women's sport (42, 46, 47).

### 4.2 Physiology and biomarkers

The investigation of physiological aspects and biomarkers in the included studies on female trail runners revealed significant alterations from pre-race to post-race measurements, underscoring the profound systemic impact of these endurance events. A primary observation was the marked elevation in biomarkers indicative of muscle damage, such as creatine kinase, which was found to increase substantially immediately post-race and often remained elevated for up to 48 h (30, 32–34). Similar elevations were also reported in other markers, such as myoglobin, which further highlights the intense physical stress and muscle tissue disruption imposed by trail races. These findings emphasize the need for tailored preparation and recovery strategies to mitigate muscle damage and facilitate restoration in female athletes (32, 33). Furthermore, the systemic inflammatory response to prolonged exertion was evidenced by elevated levels of proinflammatory cytokines, such as IL-6 and TNF-α (30, 32), further contextualizing the physiological toll of these events and reinforcing the importance of a comprehensive post-race recovery strategy. These responses underscore the systemic stress of ultramarathon events and highlight the importance of recovery strategies.

Hormonal factors appear to modulate these damage responses. Collado-Boira et al. (32) reported that higher pre-race testosterone levels in amateur female trail runners were associated with an attenuation in post-race strength loss and reduced markers of muscle damage. This study was also one of the few to document participants' hormonal contraceptive use, a crucial variable for interpreting hormonal influences on performance and physiological responses in female athletes.

In terms of muscle fiber recruitment, findings such as elevated post-race levels of slow muscle myosin suggest a significant reliance on type I (slow twitch) muscle fibers during ultramarathons (28). This predominant utilization of type I muscle fibers likely reflects an enhanced dependence on energy-efficient metabolic pathways, a critical adaptation for sustained performance in ultradistance events (48). This contrasts with shorter endurance events, where factors such as VO_2max_ are often considered more critical determinants of performance (49). Indeed, this review noted a VO_2max_​ range of 53.1 to 54.5 mL·kg^−^¹·min^−^¹ in the female trail runners, which, while competent, may not be considered exceptionally high when compared against other endurance athletes (13). As previously noted, this range could be attributed to the marked heterogeneity of participants in trail running events, with amateur and elite athletes frequently competing in the same events (29). Future research should endeavor to stratify participants by competitive level and other relevant physiological performance parameters to enhance the interpretability of findings.

Another pertinent physiological finding relates to iron metabolism. Serum ferritin, a key indicator of the body's iron stores, was reported to decrease significantly in adolescent runners in one of the foundational studies in this area (50), a finding that remains relevant for endurance athletes today. Iron status, and by extension serum ferritin levels, is particularly critical for female endurance athletes due to factors such as menstrual blood loss and the increased metabolic demands of prolonged exercise (17). Suboptimal ferritin levels, even without overt anemia, can impair aerobic performance, reduce muscular efficiency, and prolong recovery periods (51). This makes iron status an important consideration in female trail runners, especially given its association with components of the female athlete triad and relative energy deficiency in sport (RED-S) (52, 53). Future research should therefore prioritize investigating the interplay between iron status (via ferritin and other markers), menstrual cycle characteristics, and energy availability, aspects previously highlighted in the scientific literature concerning female athletes (54), to develop targeted strategies for optimizing health and performance in this group.

Despite these insights, the physiological (and particularly the hormonal) responses of female trail runners remain significantly underexplored. This is partly due to the limited detailed reporting of female-specific data in some of the broader endurance literature, and critically, within the trail running studies themselves, very few accurately documented the hormonal status (e.g., menstrual cycle phase and hormonal contraceptive type and usage) of their female participants. Given the well-established influence of ovarian hormones on numerous physiological systems relevant to exercise performance and health in women (55), this omission represents a substantial gap. It is imperative that future studies systematically collect and report data on menstrual status and hormonal contraceptive use, adhering to established guidelines (42, 46), to enable a more nuanced understanding of female physiology in the demanding context of trail running.

### 4.3 Nutrition and body composition

Optimal nutritional strategies and body composition are essential for both performance and health in trail running. This review identified six studies focusing on nutrition and body composition, which examined aspects such as feeding strategies, carbohydrate (CHO) requirements, and changes in body composition within various ultramarathon contexts. However, detailed in-race diary assessments remain scarce, limiting insights into real-time fueling practices among female trail runners. For instance, only two publications described complete dietary intake during an ultramarathon trail race (37, 38). These studies reported a significant positive correlation between higher CHO intake and faster running speeds, a finding consistent with the established role of CHO as a primary fuel source in prolonged endurance exercise (56, 57). Interestingly, these studies found no significant association between maximal blood glucose concentrations and running speed, suggesting that the consistent availability and timely supply of CHO, rather than peak circulating glucose levels *per se*, may be more critical for performance. Given the scarcity of comprehensive dietary data specific to female trail runners, future research should prioritize detailed analyses of energy and macronutrient intake (including type, amount, and timing) during diverse trail running events, as well as daily nutritional practices.

Regarding body composition, Melo et al. (36) provided valuable cross-sectional descriptive data on the anthropometric and body composition parameters of elite Colombian female trail runners. This was the only study in the present scoping review to specifically assess this relationship in elite females. Their findings suggested that female runners with a relatively low BMI might exhibit certain physiological and competitive advantages. However, this observation warrants careful consideration. While a lean physique may be beneficial for thermoregulation and running economy, an excessively low BMI [often a consequence of low energy availability (LEA)] can precipitate serious health issues, including hormonal dysregulation (e.g., menstrual dysfunction), compromised bone health, and ultimately, impaired long-term athletic development and performance (20, 52). This aligns with the broader understanding of RED-S, a syndrome that adversely affects numerous physiological functions (58). Future studies on body composition in female trail runners should therefore incorporate assessments of energy availability and health status.

Comparisons across the included studies further highlighted that elite or faster trail runners tended to present with lower body mass and BMI compared to less competitive or slower counterparts (27, 31, 35). For example, Hoffman (24) observed that top-five finishers had lower BMI values than slower runners or those who did not finish. While these cross-sectional observations are common in endurance sports, it remains challenging to discern whether such differences in body composition are a determinant of higher performance or a consequence of rigorous training and competitive success. This ambiguity underscores the need for longitudinal research that tracks changes in body composition alongside performance metrics and training load.

Preliminary evidence from this review also indicates that participation in trail running, particularly ultramarathon events, may induce alterations in body composition, affecting muscle and fat tissues (31). However, the current body of evidence is insufficient to fully elucidate the nature, magnitude, and timeline of these changes in female athletes. Longitudinal studies are therefore essential to better understand how body composition adapts across training cycles and in response to challenging races. Such research is crucial for determining appropriate energy and nutrient requirements to support training adaptations, prevent health complications associated with LEA, and ultimately optimize performance in female trail runners.

### 4.4 Injuries

Injuries were addressed in only four studies encompassing a total pool of 1,042 athletes, which reported a 32% incidence rate in female trail runners. While some individual studies, particularly those focused on shorter mountain races, reported relatively low injury rates (39), the overall findings suggest that injuries are a commonly and clinically relevant concern for female trail runners. Consistent with broader running injury literature, most injuries in female trail runners were reported to occur in the lower limbs (40, 41, 59). However, only one of the included investigations was conducted longitudinally over five seasons, providing valuable insights into injury patterns over time, and noted a substantial number of injuries (39).

Among the four injury-focused studies in female trail runners, considerable heterogeneity in injury definition and classification was evident. Future studies should aim to unify criteria for defining, recording, and reporting injuries to allow for better comparability across studies. Furthermore, such research must report on athletes' competitive levels, training backgrounds (volume, intensity, type of terrain), and running experience, as these factors are crucial for contextualizing injury risk. Standardized injury surveillance methodologies, as advocated in sports epidemiology (60, 61), would greatly advance the field.

An important finding from this review is the apparent absence of investigation into the links between injury patterns and key aspects of female athlete health, such as LEA, menstrual cycle disruptions, and how these interrelate with performance outcomes in the specific context of trail running. The consequences of LEA, often manifesting as RED-S, are well-documented in other athletic populations (58). These include increased risk of bone stress injuries, endocrine dysfunction, and impaired physiological function. However, such relationships have not been explored within the unique demands and environmental exposures of female trail runners among the studies included in this review.

Addressing this significant knowledge gap should be a priority for future research. Prospective, longitudinal studies are needed to investigate the multifactorial etiology of injuries in female trail runners, with a specific focus on the interplay between biomechanical factors, training load management, nutritional status (particularly energy availability), and endocrine health (including menstrual function and hormonal contraceptive use). Understanding these potential associations would enable the development of targeted injury prevention strategies and improve the health and well-being of female trail running athletes (62).

### 4.5 Performance

Performance in trail running events is typically quantified through race completion times or average running speeds. This scoping review identified a limited number of studies (*n* = 3) that focused on performance in female trail runners, including one study specifically investigating sex- and age-related differences in running velocity during trail races.

One study demonstrated a positive correlation between performance and specific strength measures, such as isometric maximal voluntary contraction (MVC) and ankle reactive strength, suggesting that greater strength capacities are associated with better outcomes in demanding trail running events (26). While the beneficial relationship between strength and endurance performance is well-established across numerous other sports (63), and the importance of strength training for endurance athletes is increasingly recognized (64), the investigation by Martinez-Navarro et al. (26) was the only one of the three performance domain studies to consider this association in the context of female trail running performance.

Notably, there was limited information regarding the training processes and load management strategies employed by female trail runners. The single study addressing strength levels, and the absence of published research within this review detailing training methodologies or load management specifically for this athletic group, contrasts with the more extensive literature on their male counterparts (65). This knowledge gap hampers the development of evidence-based training guidelines tailored to the unique physiological and biomechanical characteristics of female trail runners. Consequently, future research should prioritize reporting and analyzing the training practices undertaken by female trail runners of different competitive levels and age groups. Suggested variables to document would include volume, intensity, periodization, specific training modalities such as strength training, and load monitoring strategies (66). Such research would advance our understanding of performance determinants of female trail runners and allow for the optimization of specific training to enhance performance in this population.

Lastly, in the limited performance literature, the fastest finishing times were reported by runners aged over 35 years old. This phenomenon has been observed in other endurance disciplines (9, 67, 68), and the idea is that, through training, in endurance sports, especially long-distance ones, performance levels can be maintained at a high and constant level even at ages close to 50 (25). Despite this, this review shows that the absence of detailed longitudinal data tracking performance trajectories in different age cohorts of trail runners represents a significant limitation in current literature.

### 4.6 Limitations and future perspectives

Despite aiming for comprehensive coverage, this review was inherently limited by the heterogeneity of study designs and outcome measures in the existing literature. This variability likely stems from the inherent logistical challenges of conducting field-based ultramarathon research, which often leads to small, convenience-based sample sizes and difficulties in standardizing protocols across different events and environments. These constraints, coupled with the historically lower participation of women in these events, contribute to the fragmented nature of the current evidence base and explain many of the inconsistencies observed. The body of research on female trail runners had widespread inconsistencies in the reporting of participant and race characteristics, a lack of detailed female-specific physiological data, insufficient reporting of menstrual cycle status and hormonal contraceptive use, and predominantly used cross-sectional designs as opposed to longitudinal studies. These limitations impaired the possibility of synthesizing data from the included scoping review studies into generalizable recommendations. As this present study was a scoping review, we did not perform a meta-analysis. However, the identified heterogeneity highlights the value of future synthesis once reporting becomes more standardized.

### 4.7 Conclusions

This scoping systematic review demonstrates that although research on female trail runners is expanding, its practical application remains constrained by reporting inconsistencies, particularly regarding female-specific variables. Future studies should adopt more rigorous and standardized methodologies, adhering to established reporting guidelines such as PRISMA and STROBE, while also incorporating guidelines specifically designed for research on female athletes. For example, consistent tracking and verification of menstrual cycle status should be prioritized, using simple calendar tracking or, where possible, serum confirmation of hormonal profiles. In addition, standardized reporting of training load (e.g., volume, intensity, periodization) and detailed descriptions of race conditions (e.g., environmental fluctuations and terrain characteristics) are crucial for contextualizing study findings and enabling cross-study comparisons. The adoption of these practical standards is essential to build a cohesive and reproducible evidence base. Ultimately, methodologically rigorous, longitudinal studies that accurately report hormonal status, energy availability, injury risk, and training methodologies are required to optimize the health, well-being, and athletic potential of female trail runners.

## Data Availability

The original contributions presented in the study are included in the article/Supplementary Material; further inquiries can be directed to the corresponding author.

## Glossary

CSA: cross-sectional area
CSP: Castelló Penyagolosa
EMG: electromyography
FABP: fatty acid-binding protein
FEV: forced expiratory volume
FS: fractional shortening
FVC: forced vital capacity
GFR: glomerular filtration rate
HG: hand grip
HRR: heart rate reserve
IMVC: isometric maximal voluntary contraction
ITRA: International Trail Running Association
IVRT: isovolumic relaxation time
LPS: lipopolysaccharide
LVEDV: left ventricular end-diastolic volume
LVESV: left ventricular end-systolic volume
MDA: malondialdehyde
MIP: maximal inspiratory pressure
MUM: mountain ultra-marathon
PEF: peak expiratory flow
PP: per protocol
RMS: root mean square
SM: slow myosin (Note: refers to the “slow fiber-specific” context in Carmona et al. (28)/Table 2)
SV: stroke volume
SVI: stroke volume index
UTMB: Ultra-Trail du Mont-Blanc
